# Reducing stress and promoting well-being in healthcare workers using mindfulness-based cognitive therapy for life

**DOI:** 10.1016/j.ijchp.2021.100227

**Published:** 2021-02-18

**Authors:** Clara Strauss, Jenny Gu, Jesus Montero-Marin, Adrian Whittington, Cavita Chapman, Willem Kuyken

**Affiliations:** a School of Psychology, University of Sussex, United Kingdom; b Research Department, Sussex Partnership NHS Foundation Trust, Sussex Education Centre, Mill View Hospital, United Kingdom; c Department of Psychiatry, University of Oxford, Warneford Hospital, United Kingdom; d Health Education England, Kent, Surrey and Sussex, United Kingdom

**Keywords:** MBCT-L, Mindfulness, Stress, Healthcare workers, Experiment, MBCT-L, Atención plena, Estrés, Trabajadores sanitarios, Experimento

## Abstract

Healthcare workers play a critical role in the health of a nation, yet rates of healthcare worker stress are disproportionately high. We evaluated whether mindfulness-based cognitive therapy for life (MBCT-L), could reduce stress in healthcare workers and target a range of secondary outcomes. *Method*: This is the first parallel randomised controlled trial of MBCT-L. Participants were NHS workers, who were randomly assigned (1:1) to receive either MBCT-L or wait-list. The primary outcome was self-reported stress at post-intervention. Secondary variables were well-being, depression, anxiety, and work-related outcomes. Mixed regressions were used. Mindfulness and self/other-compassion were explored as potential mechanisms of effects on stress and wellbeing. *Results*: We assigned 234 participants to MBCT-L (*n* = 115) or to wait-list (*n* = 119). 168 (72%) participants completed the primary outcome and of those who started the MBCT-L 73.40% (*n* = 69) attended the majority of the sessions. MBCT-L ameliorated stress compared with controls (*B* = 2.60, 95% CI = 1.63‒3.56; *d* = -0.72; *p* < .0001). Effects were also found for well-being, depression and anxiety, but not for work-related outcomes. Mindfulness and self-compassion mediated effects on stress and wellbeing. *Conclusions*: MBCT-L could be an effective and acceptable part of a wider healthcare workers well-being and mental health strategy.

## Antecedentes/Objetivo

Los trabajadores sanitarios juegan un papel fundamental en la salud de una nación, pero sus tasas de estrés son desproporcionadamente altas. Se evaluó si la terapia cognitiva basada en mindfulness para la vida (MBCT-L) podría reducir el estrés y otras variables secundarias en trabajadores sanitarios. *Método*: Primer ensayo controlado aleatorizado de MBCT-L. Los participantes fueron asignados aleatoriamente (1:1) a un grupo MBCT-L o a una lista de espera. La variable principal fue estrés percibido (post-intervención), y las variables secundarias bienestar, depresión, ansiedad, y otras relativas al trabajo. Se utilizaron regresiones mixtas. Mindfulness y autocompasión fueron explorados como potenciales mediadores del estrés y bienestar. *Resultados*: 234 participantes fueron asignados a MBCT-L (*n* = 115) o grupo control (*n* = 119) y 168 (72%) participantes completaron la variable principal. Un 74% (*n* = 69) de quienes empezaron MBCT-L atendieron la mayoría de las sesiones. MBCT-L mejoró el estrés comparado con lista de espera (*B* = 2,60; 95% CI = 1,63‒3,56; *d* =-0,57; *p* <  0,0001). También se encontraron efectos en bienestar, depresión y ansiedad, pero no en variables del trabajo. Mindfulness y autocompasión mediaron efectos sobre estrés y bienestar. *Conclusiones*: MBCT-L podría ser una pieza eficaz y aceptable dentro de una estrategia más amplia de bienestar y salud mental para trabajadores sanitarios.

Healthcare workers play a critical role in the health of a nation and their mental health and well-being is a pre-requisite for an effective, efficient and compassionate service. Yet surveys consistently show rates of stress and mental ill health in healthcare workers are higher than in many other work settings (Weinberg & Creed, 2000). This appears to have been exacerbated by the current COVID-19 pandemic where healthcare workers have been at the frontline (Bohlken et al., 2020). In addition to the serious personal and economic consequences of high levels of stress and mental ill health in healthcare workers, these problems are associated with poor patient care and safety (Hall et al., 2016). There is therefore a need to find effective ways of ameliorating stress and promoting the well-being of healthcare workers.

Meta-analytic reviews suggest that workplace psychological approaches targeting stress, mental health and well-being can be effective, but effect sizes are typically small (Joyce et al., 2016, Rongen et al., 2013). Moreover, there are many barriers to successful implementation of such approaches, including stigma about mental health problems making some healthcare workers reluctant to seek help, high costs associated with individually-delivered interventions and clinical interventions that are not suitably adapted for the non-clinical workplace (Joyce et al., 2016). Mindfulness-based programmes (MBPs) have the potential to address these barriers by providing an approach to developing resilience by teaching foundational skills of attention, self-care, and emotional and behavioural self-regulation (Feldman and Kuyken, 2019, Pagnini et al., 2019).

MBPs teach these foundational skills across the full continuum of wellbeing, from those currently experiencing severe mental health difficulties right through to those who are flourishing (Crane et al., 2017, Huppert, 2009, López-Navarro et al., 2020, Pardos-Gascón et al., 2021). Mindfulness-Based Stress Reduction (MBSR) was developed to help people cope with stress, pain and illness (Kabat-Zinn, 1990) and Mindfulness-Based Cognitive Therapy (MBCT) integrated MBSR and cognitive-behavioural therapy to help people with a history of recurrent depression learn skills to stay well (Segal et al., 2013). MBCT for Life (MBCT-L) draws on psychological science to articulate a theoretical map and route map that speaks to the foundational skills (Feldman & Kuyken, 2019) and the curriculum is adapted from MBCT to be applicable to the general population, promoting mental health and supporting well-being more broadly (Kuyken et al., 2019).

Mindfulness-based cognitive therapy for life (MBCT-L) integrates aspects of cognitive-behavioural therapy (CBT) and MBCT specifically adapted to the general population. It focuses both on resilience in the face of stress but also more broadly on supporting well-being, making it well suited in general population workplace settings. Whilst there is growing evidence that MBPs improve stress, mental health and well-being in occupational settings (Bartlett et al., 2019) including healthcare workers (Lomas et al., 2018), there is a specific need to evaluate MBCT-L given its adaptation for the general population. Moreover, although there are promising expectations that MBPs work through the proposed mechanism of action by teaching foundational mindfulness skills (Gu et al., 2015), this is yet to be explored in occupational settings.

In summary, while the rationale for targeting stress, well-being and mental health in healthcare workers is compelling there are some important gaps in our knowledge. The effectiveness of MBCT-L, specifically designed for the general population and potentially more suitable for healthcare workers than MBPs designed for mental/physical health populations, is yet to be established, as are its potential mechanisms of action. In this paper we report on a randomised controlled trial (RCT) comparing MBCT-L with a wait-list (WL) control for healthcare workers that addresses these gaps. Our primary hypothesis is that MBCT-L will be more effective than WL in reducing stress. Secondary hypotheses address MBCT-L’s: (1) acceptability; (2) effects on wellbeing (foregrounded above other secondary outcomes given MBCT-L’s intention to enhance wellbeing); (3) effects on depression and anxiety; (4) effects on work functioning, including burnout, presenteeism and sickness absence; and finally (5) if participants learned foundational skills of mindfulness and compassion and whether effects on stress and well-being outcomes were mediated through learning these skills.

## Method

The study design and procedure are detailed in the published study protocol (Strauss et al., 2018). This study is one of two separate superiority RCTs for healthcare workers comparing (1) MBCT-L with WL and (2) CBT with WL – with separate WLs for each study. The two studies were advertised together with participants able to choose their preferred study. This paper reports on the RCT comparing MBCT-L with WL.

### Participants

Participants were healthcare workers in one of four NHS organisations in the South of England. Each healthcare organisation employed between 2,500 and 5,000 workers. To be eligible, participants had to: (1) be employed by (or working in an honorary/voluntary capacity for) one of the four NHS organisations, (2) be currently in work (i.e., not currently on sickness absence), (3) have sufficient English language ability to understand intervention information and questionnaire content, and (4) be adults (aged 18 years or older). There were no exclusion criteria.

Sample size calculations were conducted using G*Power based on an expected medium between-group effect on post-intervention stress (Cohen’s *d* = 0.50) with 90% power and *α* = .05. The estimated effect is based on effects on stress reported in trials of MBPs for healthcare workers. Calculations showed 234 participants were required assuming 40% study drop-out (i.e., participants not providing complete data).

### Procedure

Recruitment took place between July-December 2017. Study information was provided online and participants gave informed consent by completing an online form and selecting their preferred intervention (MBCT-L or CBT). Those who selected MBCT-L were shown MBCT-L courses running during the intervention period (September-December 2017) and courses starting after the intervention period, the WL (from January 2018), and asked to select their preferred course from each list.

Following consent and selection of MBCT-L courses, participants were sent an e-mail containing the link to baseline assessment measures (Time 0). Upon completion of baseline measures, participants were automatically randomised to their preferred MBCT-L course either during the intervention period (intervention) or following it (WL) by Qualtrics. Immediately after completion of their course participants were asked to complete online post-intervention measures (Time 1). Participants randomised to WL were sent an e-mail asking them to complete Time 1 measures immediately after the end of the intervention course they selected and prior to their WL course starting.

Participants were randomly allocated (1:1 ratio) to receive MBCT-L immediately or after a delay (WL). Block randomisation (block size = 4) was automated using Qualtrics. Members of the research team involved in the day-to-day management of the study were blind to block size. Participants and MBCT-L trainers were aware of group allocation, but all assessments were blind as they were completed online.

The study was granted ethical approval by the Health Research Authority in in England (Project Reference: 224584). The trial registration can be found at http://www.isrctn.com/ISRCTN11723441?q=&filters=&sort=&offset=3&totalResults=15901&pag. The trial was conducted and reported in accordance with the Declaration of Helsinki and CONSORT guidelines (Schulz et al., 2010).

Mindfulness-Based Cognitive Therapy for Life (MBCT-L; Kuyken et al., 2019) is an adaptation of the original MBCT programme which was developed for people with a history of recurrent depression at risk of depressive relapse (Segal et al., 2013). It is an 8-week, group-based (up to 15 participants), participatory psycho-educational programme that integrates CBT strategies with mindfulness practice. Each weekly session is two hours long and participants are invited to complete approximately 40 minutes per day of mindfulness practice and other home tasks. Participants learn strategies and practices: to stabilize attention; regulate their emotions and behaviours; enhance self-care and; transfer this learning into their professional and personal lives. Content in the sessions includes guided mindfulness practices, weekly homework and teaching/discussion. Please see Kuyken et al. (2019) for further details of the programme.

The MBCT teachers completed MBCT and MBCT-L teacher training and all met good practice criteria set out by the UK Network of Mindfulness-Based Teacher Training Organisations. Supervision was provided on at least three occasions per group.

### Instruments

Outcome measures were administered at baseline and post-intervention using Qualtrics. Demographic data were recorded at baseline only, and engagement measures were administered post-intervention.

Attendance at sessions was recorded by MBCT-L teachers. At post-intervention, MBCT-L participants were asked to report: (1) average number of days/week engaged in a guided mindfulness practice, not including group sessions (0–7); (2) on those days, average number of minutes per day of mindfulness practice; (3) ability to bring mindfulness principles into daily life (0–5); (4) ability to participate in MBCT-L sessions (0–5); (5) belief in effectiveness of mindfulness in helping to manage stressful situations (0–5); (6) difficulty in finding time to engage in between-session practices; (7) satisfaction with the teacher (0–5), and (8) levels of comfort with other group members (0–5). The 0–5 rating scales were anchored by *not at all* (0) and *extremely* (5).

Stress at post-treatment was the primary outcome. It was assessed with the 7-item stress subscale from the Depression, Anxiety, and Stress Scale (DASS-21; Lovibond & Lovibond, 1995). This subscale measures the presence of core stress symptoms over the past week. Responses were given on a 4-point Likert-type scale, ranging from 0 (*never*) to 3 (*almost always*). Cronbach’s alpha values were T1: α = .85; T2: α = .85.

Mental wellbeing was measured using the 7-item Short Warwick Edinburgh Mental Wellbeing Scale (SWEMWBS; Stewart-Brown et al., 2009). The SWEMWBS involves items asking experiences over the past two weeks on a 5-point Likert scale ranging from 1 (*none of the time*) to 5 (*all of the time*). Scores were transformed to the scale of the long version of the questionnaire (Stewart-Brown et al., 2009), with T1: α = .87; T2: α = .90.

Depression and anxiety symptoms were measured using the corresponding subscales from the DASS-21 (Lovibond & Lovibond, 1995). The depression subscale showed T1: α = .89; T2: α = .90; while the anxiety subscale T1: α = .78; T2: α = .81.

Burnout was measured using the Maslach Burnout Inventory-Human Services Survey (MBI-HSS; Maslach et al., 1996). The MBI-HSS consists of three subscales: emotional exhaustion, depersonalisation, and personal accomplishment. Participants were asked about the frequency with which they have experiences related to the subscales and items were answered on a 7-point Likert scale, ranging from 0 (*never*) to 6 (*every day*), being that emotional exhaustion (T1: α = .92; T2: α = .92); depersonalization (T1: α = .77; T2: α = .72); personal accomplishment (T1: α = .81; T2: α = .80).

Presenteeism was measured using the Institute for Medical Technology Assessment Productivity Cost Questionnaire (iMTA PCQ; Bouwmans et al., 2015), in its question that assess attending work while unwell (recall period 4 weeks): “how many days at work were you bothered by physical or psychological problems?”. Sickness absence was measured using the following self-report question: “Approximately how many days have you been absent from work due to sickness in the last three months?”.

Compassion for self and others was measured using the Sussex-Oxford Compassion Scales (SOCS; Gu et al., 2020). The SOCS includes two dimensions, compassion for self (SOCS-S) and others (SOCS-O). Participants indicate how true each statement is using a 5-point Likert-type scale, ranging from 1 (*not at all true of me*) to 5 (*always true of me*), obtaining SOCS-S α = .92 (T1), α = .93 (T2), SOCS-O α = .88 (T1), α = .90 (T2).

Mindfulness was measured using the Five Facet Mindfulness Questionnaire-Short Form (FFMQ-SF; Gu et al., 2016). We used the four-factor hierarchical structure without the “observing” facet, as it has been recommended in non-meditator samples (Gu et al., 2016). Items are rated on a 5-point Likert scale, ranging from 1 (*never or very rarely true*) to 5 (*very often or always true*). Cronbach’s alpha values were T1: α = .77; T2: α = .85.

### Statistical analysis

We describe participants’ characteristics at baseline by frequencies (%), medians (inter-quartile range, *IQR*), or means (*SD*), depending on the distribution of each variable. The acceptability of MBCT-L was explored through levels of engagement and self-reported satisfaction. A missing-values analysis of stress and well-being were developed using the Little’s test for missing completely at random (MCAR) data patterns including all indicators.

To explore the effectiveness of MBCT-L we conducted a between-group analysis on an intention-to-treat (ITT) basis with stress as a continuous variable. It involved a mixed-effects regression model, including time as an independent variable, and participants and sub-groups of delivery as random effects. The restricted maximum likelihood method was used, which produces unbiased estimates when using unbalanced data. Unstandardized slopes for the ‘group x time’ interaction were estimated. The same analytical strategy was used for the secondary outcomes. Effect sizes (*ES*s) were calculated using Cohen's *d* from raw data by the combined *SD* weighing the difference in the pre-post means. ESs are considered small when *d* = 0.20, medium when *d* = 0.50, and large when *d* = 0.80.

Further exploratory analyses of effectiveness on stress and well-being were carried out to estimate the complier average causal effect of treatment (CACE). This evaluates the adjusted effect of the intervention when considering participants who engaged meaningfully in MBCT-L (attended ≥ 4 sessions). Compliers were only observed among those randomised to receive the MBCT-L programme, as WL participants did not have access to the MBCT-L. A new latent variable was created to identify the compliance status of all participants from those covariates that were significant predictors of compliance in the MBCT-L group. The CACE estimation was calculated using the maximum likelihood expectation-maximisation algorithm for mixture models. *ES*s comparable to Cohen’s *d* were calculated using the formula *Δ =*β/σ, where β is the treatment effect and *σ* is the *SD* of the outcome.

Effectiveness was also explored using the Jacobson and Truax method (1991) on stress and well-being. This criterion was used to calculate absolute risk reduction (ARR), number needed to treat (NNT, 95% CI) and reliable improvement, alongside reliable deterioration as a measure of possible harm effects.

We examined whether the effect of MBCT-L on stress and well-being was mediated through changes in mindfulness and compassion. First, simple mediating models were explored by examining potential correlations between pre-post change scores of the outcomes and process variables. We then calculated the direct and indirect relationships between the treatment condition (independent variable), mindfulness, self-/others-compassion (mediators), and stress and wellbeing (dependent variables) using path analyses. We calculated the statistical power of parallel mediation models, including all significant simple mediators at the same time, by using a Monte Carlo based estimation with 10,000 replications for indirect effects (*IE*s). Regression coefficients (*B*) of bias-corrected bootstrapped *IE*s were calculated as well as their 95% CIs based on 10,000 bootstrap samples. This test overcomes problems of asymmetry in the distribution of IEs (Lockhart et al., 2011), which are statistically significant when their 95% CI does not include zero.

Analyses were carried out using STATA v12, Mplus v8.4 and SPSS v26. All the tests were bilateral with a significance level of α < .05.

## Results

**Figure 1:**
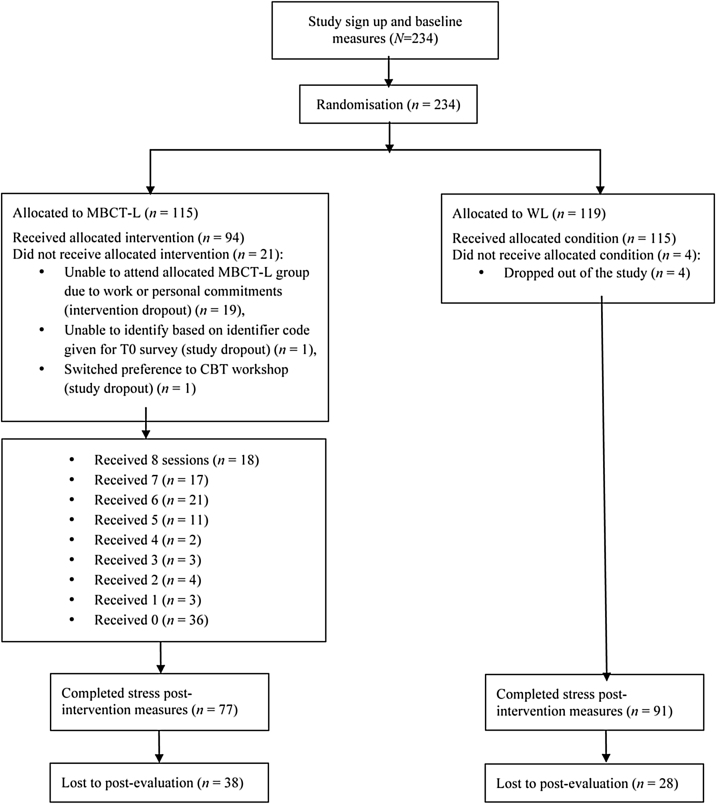
Participant flow.

As shown in Figure 1, of the 234 participants, 115 were randomised to MBCT-L and 119 to WL. Twenty-one (18%) people in the MBCT-L group withdrew from the study before starting the intervention; while 4 (3%) participants in the WL group did not receive the allocated condition. MBCT-L participants attended an average of 4.10 (*SD* = 3.19; *median* = 5; *IQR* = 0‒7) sessions. A total of 69 participants (60% of MBCT-L) attended ≥ 4 sessions. Participants who completed engagement questions (*n* = 70) reported engaging in a mindfulness practice at home on an average of 3.32 days/week (*SD* = 1.95; *median* = 3; *IQR* = 2‒5) and on these days, they reported engaging for an average of 25.92 minutes/day (*SD* = 12.63). Descriptive on the engagement ratings are presented in Table 1.

**Table 1:** Participants’ ratings of the acceptability of and engagement with MBCT-L (*n* = 70).

Items	*M(Range 0-5)*	*SD*
Ability to bring mindfulness into daily life	3.36	0.90
Ability to participate in MBCT-L sessions	3.99	1.11
Belief in effectiveness of mindfulness to manage stress	3.90	0.85
Difficulty in finding time to engage in mindfulness practices	3.60	1.15
Satisfaction with the mindfulness teacher	4.53	0.85
Comfort with other group members	4.33	1.11

The ratio of study dropouts was very similar in the two groups; 79 (69%) participants in the MBCT-L arm and 91 (77%) WL participants completed stress and wellbeing post-intervention measures. There were no significant relationships between missingness at post-treatment and variables at baseline. A missing-values analysis revealed that stress and wellbeing met criteria for MCAR [Little’s MCAR *χ^2^*(*df* = 71) = 58.34, *p* = .859].

Baseline characteristics were similar between groups (Table 2). Raw descriptive statistics and results of the between-group analyses according to the mixed regression models can be seen in Table 3. MBCT-L was significantly more effective than WL at improving stress, with moderate-large effects (*B* = 2.60; *p* < .0001; *d* = −0.72); and wellbeing, with large effects (*B* = −2.74; *p* < .0001; *d* = 0.92). MBCT-L was significantly more effective than WL for reducing anxiety, with small effects, and depression, with moderate-large effects (Table 3). For the remainder of the secondary outcomes (e.g., burnout, sickness absence and presenteeism), no significant differences between groups were observed (Table 3). For the mediating variables, MBCT-L was significantly more effective than WL at improving mindfulness and self-compassion, with moderate effects. There was no significant between-group difference in other-compassion (Table 3).

**Table 2:** Baseline characteristics of participants.

Variable	MBCT-L (*n* = 115)		WL (*n* = 119)	
	*M*	*SD*	*M*	*SD*
Age (years)	42.95	10.05	44.92	10.68
Gender	*n*	*%*	*n*	*%*
Male	20	18	18	15
Female	93	82	101	85
Ethnic group				
White	103	90	107	90
Other	12	10	12	10
Marital status				
Single	17	15	22	19
Long-term relationship	87	76	80	67
Separated/Divorced	10	9	16	13
Widowed	1	1	1	1
Educational qualification				
Postgraduate degree or above	48	42	41	35
Undergraduate degree or equivalent	44	38	57	48
A-level or equivalent	15	13	15	13
GCSE or equivalent	8	7	5	4
Job				
Administrative/Clerical	21	18	17	14
Doctor	7	6	3	3
Psychological Therapist	19	17	26	22
Nursing	26	23	33	28
Allied Health Professional	24	21	22	19
Others	18	16	18	15
Work week involving direct patient contact				
20% or less	11	11	23	20
21%‒40%	11	11	11	10
41%‒60%	18	18	23	20
61%‒80%	30	29	22	19
81% or more	33	32	36	31
Length of mindfulness practice				
No experience	65	57	67	57
Less than a year	20	17	23	20
1-5 years	22	19	21	18
Over 5 years	8	7	7	6
Frequency of mindfulness practice				
Not at all	64	56	64	54
Once a month or less	30	26	27	23
About once a week	16	14	14	12
Most days	5	4	14	12

*Note.* MBCT-L: mindfulness-based cognitive therapy for life. WL: wait-list controls.

**Table 3:** Descriptive statistics and between-group analyses on outcomes.

		MBCT-L		WL					
Variables	*n*	Pre-*M (SD)*	Post-*M (SD)*	Pre-*M (SD)*	Post-*M (SD)*	*d*	*ICC*	*B*	*p*
Stress	77/91	7.60 (3.58)	5.35 (3.12)	6.53 (3.92)	7.02 (3.74)	−0.72	.00	2.60	<.0001
Wellbeing	77/91	20.86 (2.97)	23.27 (3.95)	22.26 (3.43)	21.68 (3.51)	0.92	.00	−2.74	<.0001
Anxiety	77/91	3.88 (3.13)	3.08 (2.80)	3.18 (3.31)	3.43 (3.34)	−0.33	.03	0.92	.02
Depression	77/90	5.29 (4.19)	3.86 (3.96)	4.36 (3.51)	5.04 (3.93)	−0.55	.00	1.89	.0001
Emotional_Exaustion	76/91	24.72 (10.20)	23.38 (11.66)	22.68 (12.34)	23.04 (12.49)	−0.15	.00	1.53	.28
Depersonalization	76/91	4.43 (4.79)	4.00 (4.37)	4.39 (4.90)	4.32 (4.48)	−0.07	.03	0.31	.55
Personal_Accomplishment	76/91	35.49 (7.83)	35.75 (8.44)	37.37 (6.82)	36.66 (6.78)	0.13	.04	−0.57	.56
Sickness_Absence	76/91	4.95 (12.68)	3.07 (8.30)	3.32 (9.11)	2.96 (5.94)	−0.14	.01	1.01	.55
Presenteeism	77/91	5.31 (6.04)	4.36 (5.87)	4.53 (6.21)	4.46 (5.48)	−0.14	.00	0.57	.53
Mindfulness	77/91	37.13 (6.11)	40.91 (5.83)	38.40 (7.62)	38.91 (6.71)	0.47	.08	−3.16	<.0001
Self-Compassion	76/91	70.96 (8.43)	76.13 (10.05)	72.45 (11.99)	72.57 (11.15)	0.48	.02	−4.90	<.0001
Other-Compassion	79/91	83.78 (7.47)	83.97 (7.42)	84.22 (7.85)	83.24 (7.62)	0.15	.00	−0.95	.27

*Note.* Mixed regression analyses including groups of delivery and participants as random effects. MBCT-L: mindfulness-based cognitive therapy for life. WL: wait-list. Descriptive and effect sizes are raw data (results adjusted by regression). *ICC*: intra-class correlation coefficient (sub-groups of delivery).

Educational level was the only significant predictor of completing the programme, and thus it was included in the CACE model predicting the corresponding latent categorical variable of compliance status (*B* = 0.90; *p* < .0001; *OR* = 2.45). The entropy value of categorization was .80, indicating that classes were well distinguished. Classes with no less than 1% total count and high posterior probabilities (≥85%) were considered acceptable. The model included 188 observations, of which 122 (66%) were compliers. After adjusting for compliance, the MBCT-L intervention maintained a significant impact with large effects on stress (*B* = 3.60; *p* < .0001; *Δ* = −1.02) and wellbeing (*B* = −4.11; *p* < .0001; *Δ* = .94). Compared to ITT analyses, ESs were found to be larger for both stress and wellbeing.

Table 4 shows the reliable change for stress and wellbeing (*SE* of change = 2.03; reliable change criterion for stress = 3.98; reliable change criterion for wellbeing = 3.99). A total of 26 patients (34%) in the MBCT-L group and 9 (10%) in the WL group experienced a reliable decrease in stress (*χ^2^* = 14.42, *p* < .0001). Thus, the *ARR* in MBCT-L compared to WL was 24% (*95% CI* = 12–36%), with a NNT = 5 (*95% CI* = 2.80–8.60. A total of 3 patients (34%) in the MBCT-L group and 14 (15%) in the WL group experienced a reliable deterioration in stress between pre- and post-test (*χ^2^* = 6.05, *p* =  .01). A total of 29 patients (38%) in the MBCT-L group and 9 (10%) in the WL group experienced a reliable increase in wellbeing (*χ^2^* = 18.38, *p* < .0001). Therefore, the ARR in MBCT-L compared to WL was 28% (95% CI = 15–40%), with a NNT = 4 (95% CI = 2.5–6.5). A total of 3 patients (4%) in the MBCT-L group and 19 (21%) in the WL group experienced a reliable deterioration in wellbeing (*χ^2^* = 10.57, *p* = .0001). No serious adverse effects were reported.

**Table 4:** Reliable change on stress and wellbeing.

Reliable Change	RC-		RC0		RC+		TOTAL
Stress	*n*	*%*	*n*	*%*	*n*	*%*	*n*
MBCT-L	3	4	48	62	26	34	77
WL	14	15	68	75	9	10	91
TOTAL	17		116		35		
Wellbeing	*n*	*%*	*n*	*%*	*n*	*%*	*n*
MBCT-L	3	4	45	58	29	38	77
WL	19	21	63	69	9	10	91
TOTAL	22		108		38		

*Note.* RC-: reliable deterioration. RC0: indeterminate change. RC+: reliable improvement. MBCT-L: mindfulness-based cognitive therapy for life. WL: wait-list controls.

We computed bivariate correlational analyses between pre-post differences in stress and well-being, and pre-post differences in mindfulness and self-/other-compassion within the MBCT-L group (Table 5). Only path analysis models for outcomes with significant correlations with any process variable were computed (Table 6). The MBCT-L group showed significantly higher gains in mindfulness compared to the WL condition, and these gains predicted the change in stress and wellbeing. The 95% bias-corrected bootstrap *CI*s for the *IE*s on stress and wellbeing did not cross zero, indicating a mediation effect of mindfulness on stress and wellbeing. The MBCT-L group showed significantly higher gains in self-compassion vs the WL condition, and these gains predicted the change in stress and wellbeing. The 95% bias-corrected bootstrap CI for the IEs on stress and wellbeing were below zero, suggesting a mediation effect of self-compassion on stress and wellbeing. A power analysis including both mindfulness and self-compassion as parallel mediators showed that only the multiple mediation on wellbeing reached appropriate statistical power. A 95% bias-corrected bootstrap *CI* showed that the *IE*s through mindfulness (IE = 0.93; 95% CI = 0.37 to 1.64) and self-compassion (IE = 0.66; 95% CI = 0.23 to 1.14) on wellbeing did not cross zero, indicating that mindfulness and self-compassion in parallel significantly mediated the effect of group (MBCT-L vs WL). Nevertheless, according to the adjusted direct effects, other mediating variables could be present in all the models.

**Table 5:** Correlations in the MBCT-L Group Between the pre‒post changes in the process variables and outcomes (stress and well-being).

Process Variables/Outcomes		*Diff_*mindfulness	*Diff_*self-compassion	*Diff_*other-compassion
*Diff_*stress	*r*	−.40	−.27	−.08
	*p*	(.0003)	(.02)	(.51)
*Diff_*wellbeing	*r*	.59	.53	.21
	*p*	(<.0001)	(<.0001)	(.07)

*Note.* Diff: pre-post change. Increasing scores mean clinical deterioration in stress and improvement in mindfulness, and self/other-compassion. *N* = 77.

**Table 6:** Direct and indirect effects in the simple mediational models.

		Direct effects			Indirect effects	
Outcome/mediator	*R^2^*	path	*B*	*t*	path	*bootstrapped* (95% *CI*)
Stress	0.27					
Mindfulness	0.09	*a*	3.26	4.01*		
		*b*	−0.24	−5.33*	a_1_xb_1_	−1.27, −0.37
		*c'*	−1.96	−3.98*		
	0.23					
Self-Compassion	0.09	*a*	5.05	4.07*		
		*b*	−0.12	−4.05*	a_1_xb_1_	−1.07, −0.29
		*c'*	−2.12	−4.13*		
Wellbeing	0.39					
Mindfulness	0.09	*a*	3.26	4.01*		
		*b*	0.40	7.77*	a_1_xb_1_	0.59, 2.11
		*c'*	2.24	3.99*		
	0.35					
Self-Compassion	0.09	*a*	5.05	4.07*		
		*b*	0.24	6.77*	a_1_xb_1_	0.61, 1.86
		*c'*	2.37	4.05*		

*Note*. *p <  .0001. 95% CI: bias-corrected bootstrap 95% confidence interval.

## Discussion

Our findings suggest that a new mindfulness curriculum designed for the general population (MBCT-L) can ameliorate stress, anxiety and depression in healthcare workers, enhance wellbeing, mindfulness and self-compassion and is acceptable and engaging. The effect size on our primary outcome (stress) was moderately large, and on our secondary outcome of wellbeing was large – which is promising compared with other workplace interventions (Hall et al., 2016). There was no evidence of significant harm, when rates of reliable deterioration are benchmarked against other studies (Baer et al., 2019). Of those who started the programme (*n* = 94), 73% (*n* = 69) completed (as defined by attending the majority of sessions), suggesting acceptability, and rates of home practice were high and comparable to other mindfulness studies (Parsons et al., 2017). While the intervention improved stress, mental health (depression and anxiety) and wellbeing, it did not address workplace-specific outcomes of burnout, presenteeism and absenteeism.

Although this study was conducted before the COVID-19 pandemic, the impact of the pandemic on stress, wellbeing and mental health in healthcare workers highlights more than ever the need to find effective, acceptable and accessible ways of supporting the healthcare workforce to prioritise self-care and build resilience (Bohlken et al., 2020). Findings suggest that MBCT-L provides an effective, acceptable and accessible way of reducing stress and poor mental health and improving wellbeing for them. MBCT-L is a brief group intervention which makes it particularly suitable in healthcare settings where both time and money are in short supply.

Findings also suggest MBCT-L might be particularly suitable in the workplace more generally, beyond healthcare settings, because it teaches foundational skills that are applicable to people across the distribution of wellbeing. Interestingly, there were small group clustering effects, suggesting different group facilitators produce similar effects. This may attest to the quality of the MBCT-L teacher standardisation, training and supervision, and detailed teacher manual. Participants learned mindfulness and self-compassion skills, and that this mediated improvements in stress and wellbeing. This suggests that, as theorized, MBCT-L teaches foundational skills and this explains much of the change in stress and wellbeing. Wellbeing programmes such as MBCT-L that teach universal foundational skills in a highly accessible format can shift the population distribution towards reduced stress and greater wellbeing (Huppert, 2009).

No approach to stress and wellbeing, in the workplace or elsewhere, is likely to be a panacea, and MBCT-L is no exception. For example, MBCT-L had little impact on burnout, perhaps signalling that burnout requires more bespoke, targeted interventions (Hall et al., 2016) or that MBCT-L should be more specifically adapted to target burnout. Structural, systemic and individual factors, some changeable (e.g., management practices) and others less so (inherent challenges of the work), impact on the culture of an organisation and the wellbeing of workers (Stansfeld & Candy, 2006). Nonetheless, our study suggests MBCT-L has a place among other approaches.

The study had a number of limitations. First, whilst the study was open to all healthcare workers, the self-selected sample was predominantly White and female. Second, our control was a wait-list, rather than an active comparator. Third, the lack of long-term follow up limits our understanding of the extent to which mechanisms are linked and benefits are sustained over time. Future work should seek to explore generalizability to broader populations, including other workplace contexts, evaluate MBCT-L directly against other evidence-based interventions in adequately powered, well-designed studies.

In conclusion, these findings suggest that MBCT-L is effective in reducing stress and promoting wellbeing and mental health for workers in healthcare settings. However, it should be offered alongside a portfolio of evidence-based approaches both to enable choice and to provide other interventions focused on particular problems such as burnout.

## Funding and acknowledgments

This research was funded by 10.13039/501100003108Health Education England: Kent, Surrey and Sussex (HEEKSS) and by the Welcome Trust (104908/Z/14/Z and WT107496/Z/15/Z). For the purposes of open access the author has applied a CC BY Copyright license Author Accepted Manuscript version arising from this submission. Thank you very much to Nikki Pitman who supported the running of the study on a day-to-day basis with a great deal of passion, commitment and skill. We are also very grateful to the MBCT teachers who taught and/or supervised the MBCT-L courses in this study. Last but not least, we’d also like to extend our thanks to the NHS workers who took part in this study and gave their time to complete the study measures.
